# The mechanism of Vicenin-2 in ameliorating skin photoaging: involvement of m6A-modified macrophage polarization

**DOI:** 10.3389/fphar.2026.1778993

**Published:** 2026-02-19

**Authors:** Yan Zhou, Wenlong Shuai, Xinyi Peng, Ruishan Li, Meng Chen, Bowen Tan, Yunzhu Mu, Xi Duan

**Affiliations:** 1 Department of Dermatology, the Affiliated Hospital of North Sichuan Medical College, Nanchong, China; 2 North Sichuan Medical College, Nanchong, China

**Keywords:** M6A, macrophage polarization, NF-κB pathway, photoaging, Vicenin-2

## Abstract

**Background:**

Skin photoaging is primarily induced by ultraviolet radiation and is closely associated with chronic inflammation and degradation of the extracellular matrix, with an imbalance in macrophage polarization (elevated M1/M2 ratio) being a key factor.

**Methods:**

This study first conducted single-cell sequencing analysis to investigate the correlation between macrophage subtypes and photoaging. At the same time, we investigated the effects and mechanisms of the natural flavonoid Vicenin-2 on photoaging through both *in vivo* and *in vitro* experiments. *In vitro*, Vicenin-2 was applied to LPS-induced RAW264.7 macrophages; *in vivo*, a mouse model of photoaging was established using UVA/UVB irradiation, followed by topical treatment with different concentrations of Vicenin-2 cream.

**Results:**

It was found that in the photodamage model, the infiltration of pro-inflammatory M1 macrophages significantly increased, and the key regulatory factor KIAA1429 was positively correlated with the level of anti-inflammatory M2 macrophages. The results showed that Vicenin-2 significantly alleviated skin damage, improved hydration and collagen organization, and promoted the shift of macrophages from the pro-inflammatory M1 phenotype to the anti-inflammatory M2 phenotype. Mechanistically, Vicenin-2 upregulated the m6A modification regulator KIAA1429, enhanced overall m6A RNA methylation, and inhibited NF-κB pathway activation by suppressing p65 phosphorylation.

**Conclusion:**

The findings indicate that Vicenin-2 may mitigate skin photoaging by modulating macrophage polarization toward the M2 phenotype through m6A-dependent regulation of the NF-κB signaling pathway, supporting its potential as a novel topical agent for photoaging intervention.

## 1 Introduction

The skin, the biggest organ in the human body, is constantly exposed to a variety of environmental stimuli and acts as the main barrier between the organism and the outside world. It is essential to preserve general health by shielding the organism from detrimental outside impacts (Dąbrowska et al., 2018; Rittié and Fisher, 2015). Ultraviolet (UV) radiation is the leading cause of extrinsic skin aging, which results in a condition called skin photoaging, among the many elements influencing the skin’s regular physiological activities in daily life (Tang et al., 2024). Ultraviolet rays (UVR) can be categorized into long-wavelength ultraviolet A (UVA) and medium-wavelength ultraviolet B (UVB), both of which contribute directly and indirectly to the development of skin photoaging (Kaltchenko and Chien, 2025; Wlaschek et al., 2001). Photoaging affects the skin’s barrier function and raises the risk of several dermatological disorders, including actinic keratosis, basal cell carcinoma, and squamous cell carcinoma (D’Orazio et al., 2013; Matsumura and Ananthaswamy, 2004; Gonzaga, 2009). It also affects appearance, showing up as wrinkles, laxity, pigmentation, roughness, and dryness. The hazards of photoaging and related disorders have increased due to the intensification of environmental photochemical pollution and the thinning of the ozone layer. According to specific research, non-melanoma skin cancer is becoming more commonplace globally (Zhou et al., 2025). Additionally, this problem has a substantial financial impact; for example, the annual cost of skin cancer prevention and treatment to Australia’s healthcare system is about 1.7 billion Australian dollars (Gordon et al., 2022). As a result, photoaging is becoming a major global public health issue. There is significant scientific and therapeutic significance in exploring its molecular underpinnings and creating successful intervention techniques.

Photoprotection, topical medications, oral antioxidants, photoelectric therapy, and regenerative therapies are among the main approaches used today to prevent and treat skin photoaging (Kaltchenko and Chien, 2025; Tsai and Chien, 2022). Both of these strategies do, however, have certain drawbacks. For example, retinoids may increase collagen formation, but they can also have adverse effects, such as dryness and skin irritation. Procedures like chemical peels and photoelectric treatments may lead to skin irritation or allergic reactions and are often characterized by high costs and the need for multiple sessions (Pellacani et al., 2024; Siddiqui et al., 2024). In recent years, a growing body of research has reported that bioactive components from various sources—such as natural bioactive peptides, plant extracts, and stem cell exosomes—can alleviate photoaging through mechanisms like antioxidant and anti-inflammatory pathways (Fernandes et al., 2023). Vicenin-2, also known as 6,8-di-C-glucoside, is a naturally derived flavonoid compound. Its substantial anti-UVB toxicity and protective properties against photoaging have been shown in our earlier *in vitro* and *in vivo* investigations (Duan et al., 2019; Hu et al., 2025). However, further research is necessary to elucidate the precise molecular pathways underlying its protective effects.

According to recent studies, oxidative stress, DNA damage, inflammatory reactions, apoptosis, and extracellular matrix degradation are all involved in the pathophysiology of photoaging (Salminen et al., 2022). These processes are linked to several signaling molecules and signal transduction pathways (Chen Q. et al., 2022; Fan et al., 2024; Sun et al., 2024). In recent years, growing evidence highlights the critical role of macrophages in skin immunity. Different stimulating factors and environments influence macrophages and are roughly divided into two subgroups: M1 and M2. The M1 subtype exhibits pro-inflammatory functions and secretes pro-inflammatory cytokines, whereas the M2 subtype demonstrates anti-inflammatory properties and contributes to tissue repair (Hassanshahi et al., 2022). Notably, a significant increase in the M1/M2 ratio has been closely linked to UV-induced skin photoaging (Gather et al., 2022). Simultaneously, N6-methyladenosine (m6A), a reversible mRNA modification, is recognized as a key regulator in numerous physiological and pathological processes. Studies have found that m6A modifications can modulate immune responses by influencing immune cell functions, suggesting a potential role for m6A in regulating macrophage function and polarization (Cheng et al., 2022). Chromosome 8q22.1 contains the m6A regulatory factor KIAA1429, which is widely expressed in various organs. According to research, KIAA1429 plays a crucial role in the methyltransferase complex, as knockdown of it reduces m6A peaks by approximately four times compared to knockdown of the methyltransferases METTL3 or METTL14 (Zhang et al., 2022). Considering this, it is still unknown whether Vicenin-2 affects macrophage polarization to produce its anti-photoaging benefits and whether m6A-mediated pathways are involved. By examining its effects on macrophage polarization and the potential significance of methylation changes, our work aims to uncover the underlying mechanisms. We conducted both *in vitro* and *in vivo* experiments to validate and investigate the ideal concentration for a topical Vicenin-2 cream.

## 2 Materials and methods

### 2.1 Single-cell dataset analysis

#### 2.1.1 Data acquisition

The single-cell RNA sequencing dataset (GSE58915) was downloaded from the public Gene Expression Omnibus (GEO) database (https://www.ncbi.nlm.nih.gov/geo/info/datasets.html). This dataset was selected because it investigates dynamic changes in the gene expression profile of rat skin following ultraviolet irradiation. The dataset includes 6 control samples and 15 photoaging model samples. After downloading, gene annotation was performed using the corresponding GPL1261 platform probe annotation file.

#### 2.1.2 Data preprocessing and quality control

The single-cell expression matrix of GSE58915 was processed using the Seurat R package. Quality control metrics for each cell were calculated, including the number of detected genes (nFeature_RNA), total UMI counts (nCount_RNA), and the proportion of mitochondrial gene expression. Cells with nFeature_RNA >500 were retained, and outliers that deviated more than 3 median absolute deviations from the median in nFeature_RNA or nCount_RNA were removed. The remaining high-quality cells were used for downstream analyses.

#### 2.1.3 Data normalization and integration

Expression data were normalized using the LogNormalize method, scaling the total expression per cell to 10,000 followed by natural log transformation. Cell cycle scores were estimated using the CellCycleScoring function. Highly variable genes were identified using FindVariableFeatures, and confounding factors such as mitochondrial gene proportion, ribosomal gene proportion, and cell cycle scores were regressed out using the ScaleData function. Linear dimensionality reduction was performed using RunPCA, and principal components were selected for subsequent clustering and integration. To correct for batch effects, Harmony was applied to integrate expression data across samples. Finally, nonlinear dimensionality reduction was conducted using RunUMAP for cell cluster visualization.

#### 2.1.4 Cell type annotation

Cell clusters obtained from UMAP were annotated based on known cell markers and reference information from the CellMarker database, with a focus on cell types relevant to the pathological process of skin photoaging. Automatic annotation using the SingleR software was employed as an auxiliary approach. Combined with tissue-specific marker genes, the biological identities of the cell clusters were determined.

### 2.2 Experiments *in vitro*

#### 2.2.1 Cell culture

The RAW264.7 cell was cultured in DMEM supplemented with 10% fetal bovine serum (FBS) and 1% penicillin-streptomycin solution. Cells were maintained routinely in a humidified incubator at 37 °C with 5% CO_2_. The culture medium was replaced every 2–3 days, and cellular morphology and density were regularly monitored. When cell confluence reached 80%–90%, the cells were subcultured for the subsequent experiment.

#### 2.2.2 Induce polarization and intervention

To investigate the optimal concentration of lipopolysaccharide (LPS) for inducing the polarization of RAW264.7 cells towards the M1 phenotype, cells were treated with fresh complete medium containing LPS at concentrations of 25, 50, 100, 200, and 300 ng/mL for 24 h. Based on the determined optimal concentration, an M1 macrophage model was established. To explore the effect of Vicenin-2 on macrophage polarization, three groups were set up in the experiment: the normal group, the model group (LPS), and the Vicenin-2 group (LPS + Vicenin-2 80 μg/mL). Vicenin-2, with a purity of 99.93%, was procured from MedChemExpress.

### 2.3 Experiments *in vivo*

#### 2.3.1 Animal

Thirty-five 6-week-old female BALB/c nude mice (24 ± 2 g, SPF grade) were from Sibeifu company (Beijing, China). Throughout the experimental period, the animals were housed in a controlled environment maintained at a temperature of 21 °C–23 °C and relative humidity of 42%–66%, under a 12-h light/dark cycle. They had *ad libitum* access to standardized feed and water. All experimental procedures were conducted in strict accordance with the standard guidelines for animal care and were approved by the Animal Ethics Committee of the Experimental Animal Center of North Sichuan Medical College (Approval No.: NSMC2025-DW-021). The study was conducted under the authorization and supervision of the Institutional Animal Care and Use Committee of North Sichuan Medical College.

#### 2.3.2 Preparation for Vicenin-2 ointment

The oil phase components—including hard ester acid (14 g), monoglyceride (8 g), petroleum jelly (10 g), and nipa gold (0.1 g)—were combined and melted by heating to 75 °C–80 °C in a water bath, followed by filtration to remove impurities. The aqueous phase was prepared by dissolving sodium dodecyl sulfate (1.5 g) and glycerol (10 g) in a suitable volume of distilled water, and then heating to 75 °C–80 °C. Vicenin-2 was then dissolved in the aqueous phase at varying concentrations (25 mg, 50 mg, and 100 mg). Under continuous stirring and maintained water bath temperature (75 °C–80 °C), the aqueous phase was gradually incorporated into the oil phase. The mixture was brought to a final weight of 100 g with distilled water and ensured complete emulsification. Finally, the emulsion was homogenized and cooled to room temperature (25 °C) (Sarfraz et al., 2024). This procedure yielded Vicenin-2 creams at concentrations of 0.025%, 0.05%, and 0.1%, alongside a blank base cream without Vicenin-2.

#### 2.3.3 Animal model construction

After 1 week of acclimatization in a specific pathogen-free (SPF) facility, all mice were numbered and randomly allocated into seven experimental groups based on a random number table:Control group, Model group (UVB + UVA), Blank cream group, Blank cream + UV group (UVB + UVA + blank cream), Low-concentration Vicenin-2 group (UVB + UVA +0.025% Vicenin-2 cream), Medium-concentration Vicenin-2 group (UVB + UVA +0.05% Vicenin-2 cream), and High-concentration Vicenin-2 group (UVB + UVA +0.1% Vicenin-2 cream). This randomization procedure was performed independently by a researcher not involved in the subsequent irradiation and evaluation processes.

Starting from the second week, all groups except the Control and Blank cream groups received UVR exposure. The UVR source consisted of three UVA lamps (wavelength range 315–400 nm, peak 365 nm, 20 W each) and three UVB lamps (wavelength range 290–315 nm, peak 297 nm, 15 W each), positioned 20–25 cm above the dorsal skin of the mice. Before the experiment, the minimal erythema dose (MED) for the depilated dorsal skin of the mice was determined and set at 150 mJ/cm^2^ for this study. The planned UVA dose was ten times that of the UVB dose. Before each irradiation session, the ultraviolet intensity was measured using a UV intensity meter (Shenzhen Linshang Technology Co., Ltd., Shenzhen, China) after the lamps had been adequately preheated. The irradiation time for UVA and UVB was calculated based on this measured intensity and the desired dose, using the formula:

$$\text{Irradiation time }\left(s\right)=\text{Irradiation dose }\left(\text{mJ}/{\text{cm}}^{2}\right)/\text{Irradiation intensity }\left(\text{mW}/{\text{cm}}^{2}\right).$$

The mice underwent UVR exposure six times per week for eight consecutive weeks, with the total cumulative irradiation dose reaching approximately 48 MED. Thirty minutes after each irradiation session, the corresponding cream (blank or Vicenin-2 formulation) was applied evenly to the irradiated dorsal skin (approximately 4 cm^2^ at the center of the back) at a single application dose of 10 mg/cm^2^. Twenty-four hours after the final UVR exposure, the dorsal skin of each mouse was photographed with a digital camera for macroscopic evaluation. Subsequently, the mice were euthanized by cervical dislocation. The fresh dorsal skin was rapidly collected for measurement of skin hydration. The spleen was excised and subsequently weighed. Parts of the skin tissue were either fixed in 4% paraformaldehyde for histological processing or stored at −80 °C for further analysis.

#### 2.3.4 Skin moisture content, epidermal thickness, and spleen index

According to the Chinese national standard GB/T 5009.3-2010, dorsal skin samples were dried in an oven at 105 °C for 4 h until a constant weight was achieved to determine the skin water content. The hydration rate was calculated using the following formula:

$$\text{Hydration rate }\left(\%\right)=\left(m_{2}-m_{3}\right)/\left(m_{2}-m_{1}\right)\times 100\%$$

Where m_1_, m_2_, and m_3_ represent the weight of the weighing container, the container with the fresh skin sample, and the container with the dried skin sample, respectively. The prepared hematoxylin and eosin (H&E)-stained sections were observed under an optical microscope. Corresponding regions were photographed at the same magnification. ImageJ software (Version 1.54) was used to measure the thickness of the epidermis. During measurement, skin appendages such as hair follicles and sebaceous glands were avoided. For each sample section, three random fields of view were selected. Using the length measurement tool in the software, measurements were taken perpendicularly from the basement membrane to the stratum corneum. Typically, five measurement points were assessed per field of view, and the average value was calculated as a single measurement for that sample. Before the final experiment, mice were fasted for 12 h, and their fasting body weights were recorded. After euthanasia, the intact spleen tissue was excised and precisely weighed using an analytical balance. The spleen index was calculated according to the following formula: $\text{Spleen index}=\left(\text{Spleen mass}/\text{Body mass}\right)\times 100\%$ .

### 2.4 H&E, Sirius Red, and EVG staining

Mouse skin tissue samples were fixed in fixative solution for 24 h. After fixation, the tissues were dehydrated, embedded, and sectioned. Sections were subsequently subjected to H&E staining, Sirius Red staining, and Verhoeff–Van Gieson (EVG) staining. The stained sections were observed under optical microscopy or polarized light microscopy (FLEXACAM C1; Leica, DM6 B; Leica) at appropriate magnifications to evaluate the structural characteristics of the skin tissue. Meanwhile, the positive area of collagen fibers was quantitatively analyzed by using ImageJ. To ensure objectivity, all quantitative image analyses were performed in a blinded manner by a researcher who was unaware of the group assignments.

### 2.5 Immunofluorescence double staining

Following fixation with 4% paraformaldehyde, mouse skin tissues were embedded in paraffin and sectioned. The paraffin sections were deparaffinized in xylene I and xylene II (20 min each), followed by rehydration through a graded ethanol series (100%, 95%, 90%, 80%, and 70%). Antigen heat retrieval was performed by incubating the sections in preheated citrate buffer (pH 6.0), followed by natural cooling. After rinsing with TBST, the sections were permeabilized with 0.3% Triton X-100 for 20 min at room temperature, followed by blocking with 3% BSA. The sections were incubated overnight at 4 °C with the primary antibody against CD68 (28058-1-AP, 1:500, Proteintech, China), followed by washing with TBST. They were incubated in the dark at room temperature with the secondary antibody (11065, 1:1000, AAT United States) for 1 h. After rinsing the sections with TBST, repeat the antigen heat repair, block, and then incubate the primary antibody of CD86 (NBP2-25208, 1:300, NOVUS, United States) and the secondary antibody (11070, 1:1000, AAT, United States) in the dark again. Finally, nuclei were stained with DAPI (S2110, Solarbio, China). The same steps were used to complete the double-labeled immunofluorescence staining of CD68 and CD206 (32647-1-AP, 1:200, Proteintech, China). Finally, all samples were imaged using confocal microscopy (BX53, Olympus).

### 2.6 Enzyme-linked immunosorbent assay

The concentrations of IL-6 (EM0121, Fine Biotech, China), IL-10 (EM0100, Fine Biotech, China), and TNF-α (EM0183, Fine Biotech, China) in skin tissue were quantified using enzyme-linked immunosorbent assay (ELISA). Skin tissue samples were homogenized in pre-cooled phosphate-buffered saline, and the supernatants were collected for analysis. The experimental procedures were strictly performed according to the manufacturer’s instructions. The absorbance (OD value) of each well was measured at a wavelength of 450 nm using a microplate reader. The corresponding expression concentrations in the samples were calculated based on a standard curve.

### 2.7 Western blotting

Homogenize the cells and tissues in the lysis agent. The resulting suspensions were collected and subjected to ultrasonic disruption on ice. After centrifugation, the supernatant was carefully collected, and the protein concentration was determined using a bicinchoninic acid (BCA) protein assay kit. Proteins were separated by sodium dodecyl sulfate-polyacrylamide gel electrophoresis (SDS-PAGE) and subsequently transferred onto a polyvinylidene fluoride (PVDF) membrane. The membrane was blocked with blocking buffer for 1 h at room temperature, followed by an overnight incubation at 4 °C with the following primary antibodies: anti-KIAA1429 (30166-1-AP, 1:2000; Proteintech, China), anti-NF-κB p65 (10745-1-AP, 1:1000; Proteintech, China), anti- Phospho-NF-κB p65 (82335-1-RR, 1:2000; Proteintech, China), anti-β-actin (20536-1-AP, 1:4000; Proteintech, China), anti-iNOS (18985-1-AP, 1:5000; Proteintech, China), and anti-Arg-1 (16001-1-AP, 1:5000; Proteintech, China). After washing, the membrane was incubated with a horseradish peroxidase (HRP)-conjugated secondary antibody (LF102, 1:2000; Epizyme Biotech, China) for 1 h at room temperature in the dark. The immunoreactive bands were visualized using an enhanced chemiluminescence (ECL) detection reagent and captured with a chemiluminescence imaging system (Shanghai Qinxiang Scientific Instruments Co., Ltd., Shanghai, China). Band intensity was quantified using ImageJ software (Version 1.54). The protein expression levels were normalized to β-actin, which served as an internal control for loading.

### 2.8 Dot blot analysis

Total RNA was extracted from fresh skin tissue using the Trizol method. The concentration and purity of the RNA were determined using a nucleic acid concentration analyzer. The denaturation solution (a 3:2 mixture of 20× SSC buffer and 37% deionized formaldehyde) was mixed with RNA (0.5 μg) at a 1:1 ratio. The mixture was then denatured at 95 °C for 5 min in a PCR instrument. The denatured samples were spotted onto a nitrocellulose (NC) membrane. The NC membrane was cross-linked under a 302 nm ultraviolet lamp for 6 min. After cross-linking, the membrane was washed with PBST and blocked with 5% non-fat dry milk on a shaker for 2 h at room temperature. Subsequently, the membrane was incubated overnight at 4 °C on a shaker with an m6A antibody (MA5-33030, 1:1000; Invitrogen, United States). Following primary antibody incubation, the membrane was washed with PBST and then incubated with an HRP-conjugated secondary antibody (LF102, 1:2000; Epizyme Biotech, China) prepared in 5% non-fat dry milk on a shaker for 2 h at 4 °C. After further washing with PBST, the antibody binding was detected using an ECL substrate, and the signals were captured using a chemiluminescence imaging system.

### 2.9 Statistical analysis

Data is presented as the mean ± standard deviation (SD) from at least three independent biological replicates. All statistical analyses were performed using GraphPad Prism software (version 9.0.0). The normality of data distribution for each group was first assessed using the Shapiro–Wilk test. For data conforming to a normal distribution, homogeneity of variances was evaluated with the Brown–Forsythe test. When variances were homogeneous, comparisons between two groups were performed using an unpaired two-tailed Student’s t-test, while comparisons among the seven experimental groups were analyzed by one-way analysis of variance (ANOVA) followed by Tukey’s *post hoc* test. When variances were heterogeneous, Welch’s t-test was used for two-group comparisons and Welch’s ANOVA followed by the Games-Howell *post hoc* test was applied for multi-group comparisons. For data that did not satisfy the normality assumption, non-parametric tests were employed: the Mann–Whitney U test for two-group comparisons and the Kruskal–Wallis test followed by Dunn’s *post hoc* test for multi-group comparisons. A *p* value <0.05 was considered statistically significant, with significance levels denoted as*P < 0.05, **P < 0.01, ***P < 0.001. Exact p values are provided in the text when necessary.

## 3 Results

### 3.1 Single-cell sequencing analysis reveals M1 macrophage polarization in photoaged skin

**FIGURE 1:**
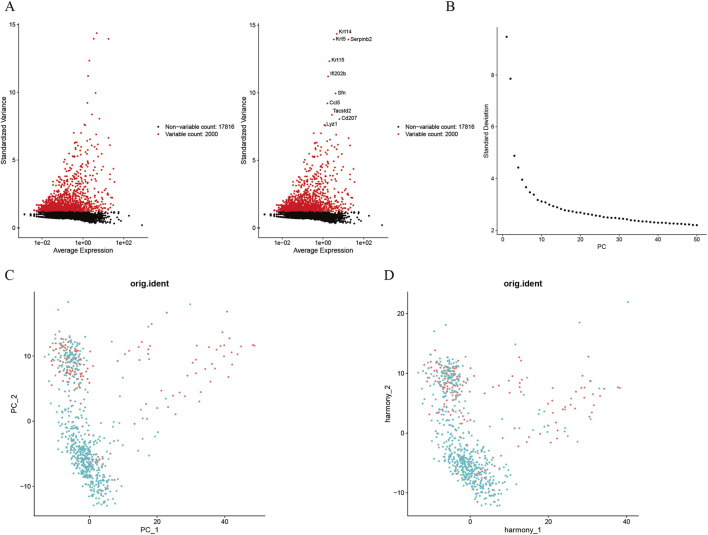
Single-cell transcriptomic data. Footnotes: **(A)** Screening of highly variable genes. **(B)** Scree plot of principal component analysis (PCA). **(C)** Visualization of PCA results. **(D)** Revisualization of double principal component analysis results after adjustment.

**FIGURE 2:**
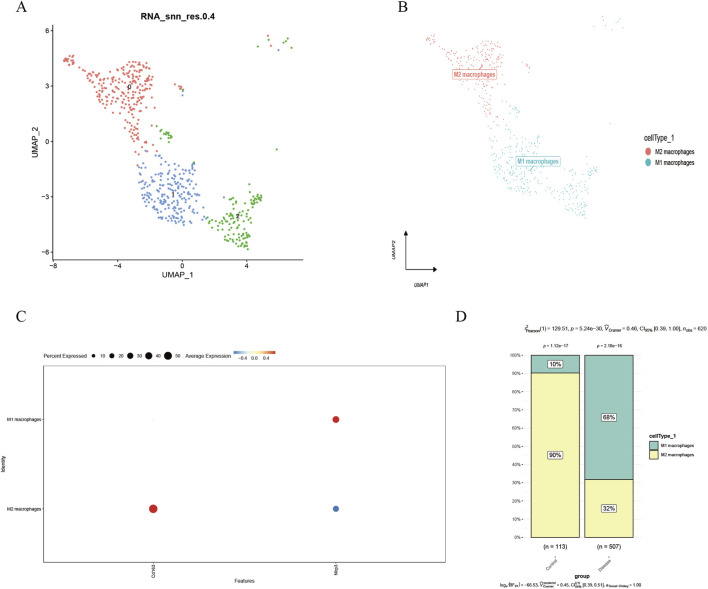
Cell type identification of single-cell RNA sequencing data. Footnotes: **(A)** UMAP visualization of cell clustering based on RNA expression profiles (resolution = 0.2). **(B)** Cell subset annotation: Macrophages were identified as M1 and M2 subtypes using characteristic markers. **(C)** Bubble plot showing expression of characteristic markers for macrophage subtypes. **(D)** Bar plot displaying the proportional distribution of cell types across sample groups.

We first identified 2,000 highly variable genes (Figure 1A), followed by sequential data processing including normalization, homogenization, principal component analysis (PCA), and Harmony integration (Figures 1B–D). After filtering out non-target cells, uniform manifold approximation and projection (UMAP) was performed for dimensionality reduction, yielding three distinct cell clusters (Figure 2A). Based on established cellular markers, these clusters were annotated as two major macrophage subtypes: M2 macrophages and M1 macrophages (Figure 2B). The expression of key marker genes for these two cell types was visualized using a bubble plot (Figure 2C), and a stacked bar chart was used to illustrate their proportional distribution across experimental groups (Figure 2D). The analysis revealed a significant increase in the proportion of M1 macrophages and a corresponding decrease in M2 macrophages within the photoaging.

### 3.2 Analysis of KIAA1429 expression

**FIGURE 3:**
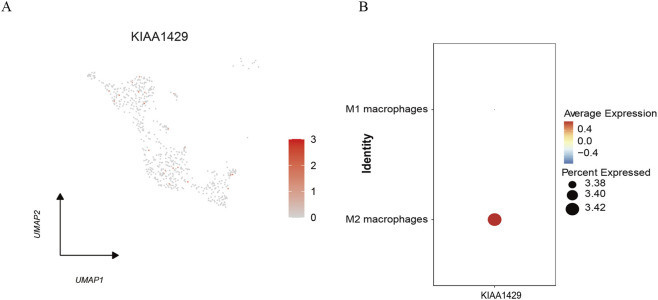
Expression distribution of KIAA1429. Footnotes: **(A)** Expression distribution of KIAA1429 in the UMAP clustering plot. **(B)** Bubble plot showing KIAA1429 expression across macrophage subsets.

We visualized the expression of the KIAA1429 gene at the single-cell level using the Dotplot function from the Seurat package (Figure 3A). The results indicated that KIAA1429 is predominantly highly expressed in M2 macrophages (Figure 3B). All analyses were conducted using standardized data and pre-annotated cell types.

### 3.3 Vicenin-2 alleviates mouse photoaging via modulation of M2 macrophages

**FIGURE 4:**
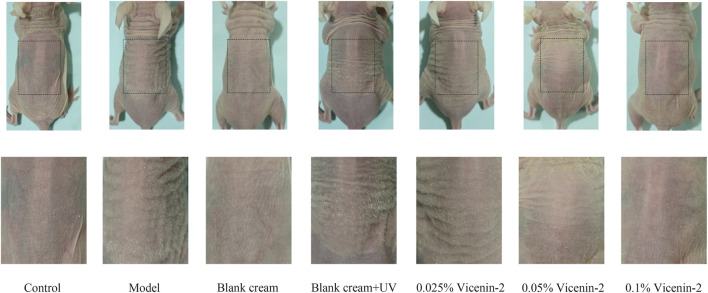
The manifestations of the skin on the back of mice.

The dorsal skin of various experimental groups showed apparent phenotypic differences when examined under a microscope. With a pink to light pink color, a smooth and fine texture, excellent elasticity, and no discernible wrinkles, hyperpigmentation, or hypopigmentation, the skin in the Control group had a normal look. The Model group (UVB + UVA irradiation), on the other hand, showed clear indications of photoaging. The skin lost its luster, became rough to touch, and developed coarse transverse wrinkles, particularly within the irradiated area. Additional observations included increased skin thickness and evident hyperpigmentation. Skin manifestations on mice’s backs in the blank cream group were identical to those in the control group, and skin manifestations in the low-concentration Vicenin-2 group were similar to those in the model group. The skin of mice in the medium and high concentration Vicenin-2 groups gradually returned to normal, with the high concentration group exhibiting a response similar to that of the normal group. For detailed visual documentation, please refer to Figure 4.

**FIGURE 5:**
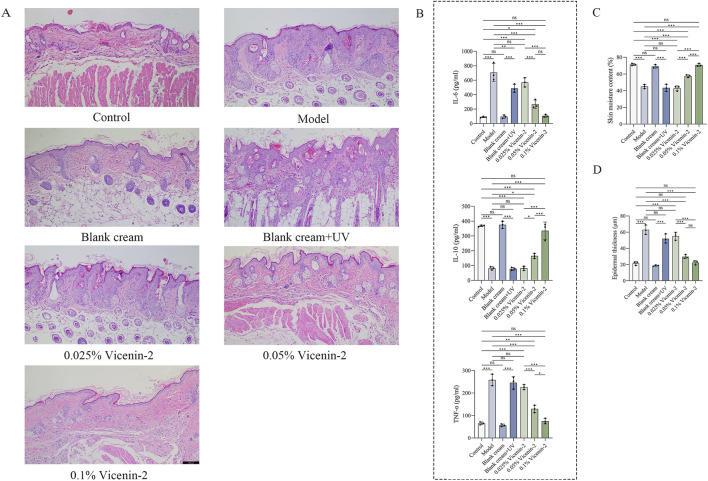
Relevant indicators for developing a model of mouse photoaging. Footnotes: **(A)** Effects of Vicenin-2 on UV-induced skin injury based on H&E staining; **(B)** The expression levels of IL-6, IL-10, TNF-α in photoaging area skins; **(C)** The skin moisture content of mice in each group; **(D)** The epidermal thickness of the skin in each group of mice. The one-way ANOVA was used for comparisons among multiple groups (n = 3). *P < 0.05, **P < 0.01, ***P < 0.001, ns: no significance.

In addition to the macroscopic observations of the dorsal skin, the successful establishment of the photoaging model was further evaluated by measuring skin hydration, epidermal thickness, and the levels of IL-6, IL-10, and TNF-α in skin tissue. Skin sections stained with hematoxylin and eosin (H&E) were histologically examined to determine overall skin morphology. In the H&E-stained sections, the skin structure of the Control group was intact, with neatly arranged cells and normal epidermal thickness; all epidermal layers were clearly defined. In contrast, the Model group (UVB + UVA irradiation) exhibited significant thickening of the stratum corneum and spinous layer, disordered cell arrangement, and infiltration of inflammatory cells such as neutrophils and lymphocytes in the dermis (Figure 5A). Quantitative analysis confirmed increased epidermal thickness and decreased skin hydration in the Model group compared to the Control group (Figures 5C,D). ELISA results demonstrated that the levels of pro-inflammatory cytokines (IL-6, TNF-α) were significantly elevated in the Model group, while the anti-inflammatory cytokine IL-10 was also increased, collectively indicating the successful induction of photoaging (Figure 5B). Observation of other groups revealed that the histological presentation of the Blank cream group was similar to that of the Control group. Quantitative analyses of inflammatory cytokine levels, epidermal thickness, and skin hydration showed no statistically significant differences between these two groups, indicating that the cream base itself had no significant irritant or toxic effects on mouse skin. The Blank cream + UV group displayed structural disorganization and epidermal thickening comparable to the Model group, with no significant differences in the quantitative indices of inflammation, epidermal thickness, and skin hydration compared to the Model group. This confirms that the blank cream base itself provided no therapeutic effect against photoaging. Among the mice treated with low, medium, and high concentrations of Vicenin-2, the Low-concentration Vicenin-2 group showed similar indicators to the Model group. However, intervention with medium and high concentrations of Vicenin-2 resulted in a gradual restoration of skin structure towards normality, characterized by reduced epidermal thickness, increased skin hydration, and alleviated inflammation. Notably, the High-concentration Vicenin-2 group exhibited the most significant anti-photoaging effects. For detailed data, please refer to Figure 5.

**FIGURE 6:**
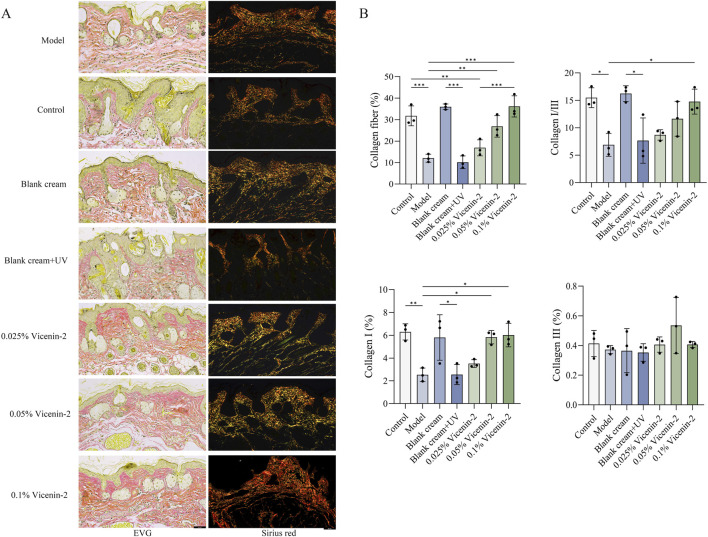
Determination of collagen fibers. Footnotes: **(A)** Effects of Vicenin-2 on UV-induced skin injury based on EVG and Sirius Red staining; **(B)** Collagen fiber content. The one-way ANOVA was used for comparisons among multiple groups (n = 3). *P < 0.05, **P < 0.01, ***P < 0.001, ns: no significance. Only display the identifiers of statistical significance.

To evaluate the content, ratio, and morphological characteristics of collagen fibers in mouse skin, EVG staining and Picrosirius Red (Sirius Red) staining were performed. In EVG-stained sections, collagen fibers were stained bright red, while elastic fibers appeared black. Sirius Red staining binds explicitly to collagen fibers. The binding of Sirius Red to collagen enhances its natural birefringence, allowing for the differentiation of collagen types under polarized light microscopy. Under polarized light, type I collagen fibers exhibit strong birefringence and appear orange-yellow or bright red, whereas type III collagen fibers appear green. In EVG staining, the collagen fiber content in the model group was significantly reduced, with a disordered arrangement, breakage, and degeneration. In contrast, the collagen in the Control group mice was neatly arranged, dense, and distributed in a wavy pattern. The Low-concentration Vicenin-2 group exhibited collagen fiber characteristics similar to those of the Model group. In contrast, intervention with medium and high concentrations of Vicenin-2 effectively promoted the recovery of collagen fibers (Figure 6A). Meanwhile, the quantitative analysis of the positive area of collagen fibers in the sections using ImageJ also indicated that the content of collagen fibers decreased in the model group and the low-concentration Vicenin-2 group. To distinguish the content of type I and type III collagen fibers, which are more commonly distributed in the skin, and the ratio of type I/III collagen fibers, we stained the sections with Sirius Red (Figure 6A). Quantitative analysis also indicated the same trend (Figure 6B). It was confirmed that low concentrations of Vicenin-2 had no significant value in the recovery of collagen fibers. In contrast, medium and high concentrations of Vicenin-2 effectively promoted the synthesis and proportion of collagen fibers, allowing them to return to normal. For detailed data and content, please refer to Figure 6.

**FIGURE 7:**
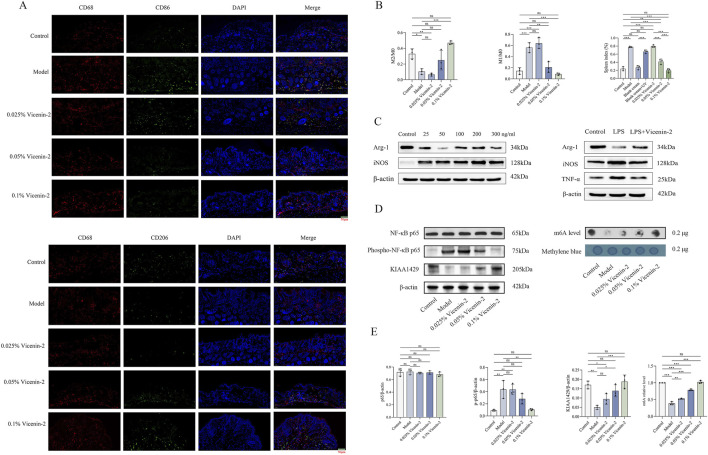
Vicenin-2 promotes the polarization of macrophages towards M2 by down-regulating the NF-κB pathway through m6A modification. Footnotes: **(A)** Immunofluorescence staining of macrophages; **(B)** Quantitative analysis of macrophage subtypes and spleen index; **(C)** The influence of Vicenin-2 on macrophage polarization *in vitro* experiments was determined by the Western blot method; **(D)** The expression of the NF-κB pathway and KIAA1429 in mice was determined by the Western blot method, and the overall m6A level was determined by the dot blot method; **(E)** Quantitative analysis of m6A modification levels (compared with the control group) and NF-κB pathway expression level. The one-way ANOVA was used for comparisons among multiple groups (n = 3). *P < 0.05, **P < 0.01, ***P < 0.001, ns: no significance.

Additionally, we stained slices of mouse skin tissue using immunofluorescence. By measuring the fluorescence intensity of macrophage-related markers (CD68, CD86, CD206), it was discovered that the proportion of M1 (CD86/CD68) in the model group was higher than that in the normal group. Still, the proportion of M2 (CD206/CD68) was lower. Meanwhile, treatment with a low concentration of Vicenin-2 did not significantly ameliorate this phenomenon, and quantitative examination of fluorescence intensity revealed no statistically significant difference compared to the Model group. However, medium and high dosages of Vicenin-2 dramatically rectified the polarization imbalance of unbalanced macrophages, reducing M1 and increasing M2 (Figure 7A). Similarly, after assessing the spleen index of mice in each group, we discovered that the model group had a considerably higher spleen index, indicating that immune cell proliferation and activation, as well as the aggravation of inflammatory responses, were amplified. After administration with an adequate concentration (medium and high concentrations) of Vicenin-2, the spleen index gradually decreased, indicating that Vicenin-2 influences the inflammation-immune response in mice (Figure 7B). All findings suggest that medium and high concentrations of Vicenin-2 decrease photoaging by correcting the imbalance in macrophage polarization. For detailed data and content, please refer to Figures 7A,B.

### 3.4 Vicenin-2 modulates macrophage polarization *in vitro*

Cell experiments also confirmed the same phenomenon. In this experiment, to establish the optimal concentration of LPS for inducing the M1 macrophage model, RAW264.7 cells were treated with various concentrations of LPS. The expression levels of macrophage subtype-specific markers were subsequently analyzed by Western blot. The results revealed that LPS treatment enhanced inducible nitric oxide synthase (iNOS) expression and suppressed arginase-1 (Arg-1) expression across some groups (Figure 7C). Notably, the most significant effect was observed at an LPS concentration of 50 ng/mL, which resulted in the lowest Arg-1 expression and a marked increase in iNOS expression. Consequently, 50 ng/mL LPS was selected for establishing the M1 model in subsequent experiments. Following the induction of M1 polarization in RAW264.7 cells via LPS stimulation, the cells were treated with Vicenin-2 (80 μg/mL). The experiment comprised three groups: a normal control group, a model group (LPS), and a Vicenin-2 treatment group (LPS + Vicenin-2, 80 μg/mL). Western blot analysis demonstrated that treatment with Vicenin-2 led to a significant reduction in the expression of tumor necrosis factor-alpha (TNF-α) and iNOS, while concurrently enhancing the expression of Arg-1 (Figure 7C). These findings indicate that Vicenin-2 alleviates LPS-induced M1 polarization and promotes a shift towards M2 polarization in RAW264.7 cells, confirming that Vicenin-2 exerts its effects by influencing macrophage polarization.

### 3.5 Vicenin-2 modulates the NF-κB signaling pathway via m6A

The NF-κB signaling pathway is essential for controlling the polarization of macrophages. We measured the expression of KIAA1429, total m6A modification levels, and the phosphorylation status of NF-κB component p65 in skin tissues to see if Vicenin-2 affects this pathway through m6A-related processes. Western blot analysis revealed that, compared to the Control group, the Model group (UVB + UVA irradiation) exhibited decreased expression of KIAA1429 and reduced global m6A modification levels, accompanied by an increased ratio of phosphorylated p65 (p-p65) to total p65. These changes were not significantly reversed by treatment with low-concentration Vicenin-2. On the other hand, KIAA1429 expression was significantly upregulated, global m6A modification was enhanced, and the p-p65/p65 ratio was significantly reduced following intervention with medium and high quantities of Vicenin-2. Notably, the high-concentration Vicenin-2 group demonstrated the most pronounced effects. These findings suggest that medium and high concentrations of Vicenin-2 cream alleviate photoaging by upregulating KIAA1429 expression, increasing global m6A modification levels, and subsequently inhibiting the activation of the NF-κB signaling pathway (Figures 7D,E). Detailed information on all quantitative data in the study can be found in the Supplementary Material.

## 4 Discussion

Skin aging is induced by the combined effects of intrinsic chronological aging and extrinsic aging, with the latter being primarily driven by photoaging (Kim et al., 2022). The detrimental effects of UVR on the skin encompass both acute damages, such as sunburn, erythema, and blistering, and chronic damage, which include photoaging, pigmentary disorders, and skin cancer. Cyclobutane-pyrimidine dimers (CPDs) and 6-4 photoproducts (6-4PPs) are the two main photoproducts that are formed when UVR directly damages DNA in the setting of chronic damage (Sinha and Häder, 2002). These pyrimidine dimers can disrupt DNA replication and repair processes, cause DNA strand breaks, and cause a variety of harmful genetic alterations (Roy, 2017; Cadet and Douki, 2018; Liu et al., 2022; Livneh et al., 1993). Secondly, UVR induces oxidative stress by generating an excess of reactive oxygen species and reactive nitrogen species, thereby disrupting the pro-oxidant/antioxidant balance within the skin (Kammeyer et al., 2015; Masaki, 2010). Thirdly, UV exposure activates multiple signaling pathways that contribute to a state of local chronic inflammation. For instance, it activates the NF-κB signaling pathway, promoting the release of pro-inflammatory cytokines such as IL-1, IL-6, and TNF-α (Bang et al., 2021). Concurrently, UVR activates the MAPK signaling pathway, leading to the formation of the AP-1 transcription factor (Jinlian et al., 2007). Furthermore, UV exposure induces the expression of matrix metalloproteinases (MMPs), including MMP-1 and MMP-3, which degrade collagen fibers in the extracellular matrix (Brenneisen et al., 2002; Oh et al., 2006). Additionally, it suppresses the TGF-β/Smad signaling pathway, resulting in reduced collagen synthesis by fibroblasts (Quan et al., 2004).

While numerous studies have focused on the mechanisms of UVR-induced skin photoaging, emerging evidence highlights that an imbalance in macrophage polarization is a critical contributing factor (Hassanshahi et al., 2022; Gather et al., 2022). The diversity and extraordinary plasticity of the monocyte-macrophage lineage are its defining features, and the plasticity of macrophages is a significant factor in chronic inflammation (Locati et al., 2020). The pro-inflammatory M1 phenotype and the anti-inflammatory M2 phenotype are the two primary subsets of macrophages that can differentiate in response to various microenvironmental stimuli. These two subtypes are alternatively active during inflammation, and tissue homeostasis depends on the M1/M2 ratio remaining roughly constant (Luo et al., 2024). M1 macrophages initiate and sustain inflammatory responses by secreting pro-inflammatory cytokines and recruiting other immune cells to inflamed tissues. In contrast, M2 macrophages phagocytose apoptotic cells, coordinate tissue integrity, and release anti-inflammatory mediators (Viola et al., 2019). Crucially, M1 macrophages have been shown to reduce type I collagen expression and accelerate collagen degradation, whereas M2 macrophages promote collagen and protein synthesis in dermal fibroblasts, thereby facilitating collagen fiber formation (Zhang et al., 2021; Horiba et al., 2023). The disruption of the M1/M2 balance induced by UVR leads to chronic inflammation, accelerating the aging of dermal fibroblasts, ultimately promoting skin aging (Oh et al., 2023; Hu et al., 2024).

In our study, to verify the changes in the polarization state of macrophages in the ultraviolet-induced photoaging model, we employed single-cell sequencing analysis. After standardizing, reducing dimensionality, and performing clustering analysis on the data, two primary subsets—M1 and M2 macrophages—were identified. The analysis revealed that in the photoaging disease group, the proportion of M1 macrophages was significantly increased, while the proportion of M2 macrophages correspondingly decreased, which is consistent with our histological findings. Additionally, by examining the expression of the key regulatory factor KIAA1429, we found that it was specifically highly expressed in M2 macrophages, further suggesting that KIAA1429 may be closely associated with the functional maintenance of the M2 phenotype. Based on the results of single-cell sequencing, we first demonstrated that Vicenin-2 could reverse LPS-induced M1 polarization of macrophages *in vitro* experiments, suggesting its potential to correct the imbalance in macrophage polarization ratios associated with chronic inflammation. Comprehensive vivo experiments followed this to validate this phenomenon. In addition to disordered skin structure, epidermal thickening, exacerbated inflammation, and collagen fiber degradation, comparisons between the photoaging model group and the normal control group showed a significant increase in the M1 proportion and a decrease in the M2 proportion. These findings confirm a strong correlation between macrophage polarization imbalance and photoaging. To further verify the *in vitro* observation that Vicenin-2 intervenes in the dysregulated macrophage polarization, we applied topical creams containing low, medium, and high concentrations of Vicenin-2 to UV-induced photoaging mice under controlled conditions. The results showed that medium and high concentrations of Vicenin-2 cream effectively alleviated photoaging by reducing the expression levels of inflammatory factors in the skin, promoting collagen synthesis, and reversing the destruction of standard skin tissue structure. Concurrently, Vicenin-2 treatment at these concentrations reversed the UV-induced imbalance in the M1/M2 ratio. However, the low-concentration Vicenin-2 cream did not elicit significant effects, likely because the amount of the active compound, Vicenin-2, was insufficient to exert a therapeutic impact. In related research, we are also considering the role of Vicenin-2, a flavonoid compound, in associated diseases. Flavonoids can alter the M1/M2 polarization of macrophages, resulting in a significant decrease in the expression of the surface marker CD86 of M1-type macrophages and a substantial increase in the expression of the M2 marker CD206. As a type of flavonoid compound, Vicenin-2 may have similarities in molecular structure and can exert similar functions.

After the above research background and experimental exploration, the specific mechanism by which Vicenin-2 regulates macrophage polarization remains unclear. The m6A modification is the most common internal chemical modification of RNA, significantly affecting various biological processes, including RNA transcription and protein expression. Numerous studies have shown that RNA m6A modification is involved in the polarization process of macrophages (Zhang et al., 2025; Wu et al., 2024). The research of Fu et al. (2025) indicates that the m6A modification of TNFSF9 can induce M2 polarization of tumor-associated macrophages. Another study demonstrated that knockdown of METTL14 inhibited the overall increase in m6A levels and the M2 polarization of macrophages (Cao et al., 2025). All suggest that the rise in m6A modification levels can promote the polarization of macrophages towards an M2 phenotype. KIAA1429 plays a key role in m6A methylation. As a scaffold that connects the catalytic core component of the m6A methyltransferase complex and plays a crucial regulatory role, Lan et al. (2019) discovered that KIAA1429 was enriched in the 3′-untranslated region and close to the stop codon of the RNA substrate in m6A modification. For regioselective m6A modification, KIAA1429 can enlist the help of the m6A methyltransferases METTL3, METTL14, and WTAP (Chen J. et al., 2022). Therefore, to explore whether Vicenin-2 interferes with the m6A modification regulated by KIAA1429 to interfere with macrophage polarization, we determined the expression level of KIAA1429 in different groups by the Western blot method and the total RNA m6A modification level by the dot blot method. In the skin tissue of mice, we found that the expression of KIAA1429 and the total m6A modification in the model group were significantly lower than those in the normal group. The expression of KIAA1429 and the total m6A modification in the mouse group treated with medium and high concentrations of Vicenin-2 were significantly higher than those in the model group. Moreover, compared with the model group, the proportion of M1 in macrophages decreased, and the proportion of M2 increased. Vicenin-2 treatment concurrently upregulated the m6A methyltransferase KIAA1429, increased global m6A methylation levels, and promoted macrophage polarization toward the M2 phenotype. This coordinated alteration suggests that these events may be interconnected within a shared regulatory axis rather than occurring independently. Therefore, we propose that Vicenin-2 likely facilitates the shift to M2 macrophage polarization by enhancing RNA m6A modification via KIAA1429 upregulation, which may represent a core mechanism underlying its anti-photoaging effects.

The m6A modification of RNA does not merely affect the expression of a specific mRNA; it influences the up-regulation or downregulation of numerous downstream proteins or signaling pathways. The research by Liu et al. confirmed that KIAA1429 regulates the m6A modification of LRP4 mRNA, thereby enhancing the stability and expression of LRP4. This enabled LRP4 to strengthen the recruitment of TNFAIP3, thereby inactivating the NF-κB signal (Liu et al., 2025). The research of Yuan et al. (2024) also confirmed that m6A modification stabilized the expression of Umod and inhibited the NF-κB and ERK signaling pathways. Similar ones were confirmed in another study (Yin et al., 2021). The NF-κB signaling pathway is a key pathway involved in inflammatory responses, and the activation of NF-κB is mainly related to the nuclear translocation of the p50/p65 heterodimer (Geiß et al., 2022). Research has shown that the M1 polarization and inflammatory response of macrophages depend on the participation of the NF-κB signaling pathway. Activating the NF-κB pathway can promote M1 polarization (He et al., 2024; Lin et al., 2020). Inhibiting the NF-κB signaling pathway can transform macrophages into the M2 phenotype (Abd-Elhalem et al., 2023). We determined the expressions of NF-κB signaling pathway-related proteins p65 and p-p65 in different groups by the Western blot method. The p65 protein did not significantly differ between the groups, but the expression of its phosphorylated form, p-p65, did. The model group’s proportion of p-p65 was higher than that of the control group, and the level of p-p65 significantly decreased following intervention with Vicenin-2 cream at appropriate concentrations (0.05% and 0.1%). This suggests that a proper concentration of Vicenin-2 can inhibit the NF-κB signaling pathway by regulating the m6A modification level, thereby promoting the transition from M1 to M2 subtypes of macrophages.

However, this study still has certain shortcomings. To begin with, the use of Vicenin-2 has only been preliminarily tested in live mice. Future studies will involve clinical trials to assess the efficacy, safety, and adverse effects of this approach in humans and primates. Another limitation pertains to the exploration of the role of m6A modification in macrophage polarization. We did not knock down or overexpress the relevant methylation modification enzymes (KIAA1429, METTL3, METTL14, WTAP, and FTO) to directly study the link between changes in M6a modification levels and distinct macrophage polarization subtypes. These shortcomings also indicate the path of our future research.

## 5 Conclusion

Our research confirms Vicenin-2’s ability to resist photoaging by interfering with macrophage polarization. Additionally, it has been shown to downregulate the NF-κB signaling pathway by promoting RNA m6A modification, which promotes macrophage polarization towards the anti-inflammatory phenotype M2. In this work, we also investigated the optimal concentration of topical Vicenin-2 cream at the animal level (0.1%), providing good experimental evidence for the subsequent clinical application of Vicenin-2 cream. In addition to traditional therapies, it also offers new concepts for alternative photoaging treatment solutions.

## Data Availability

Sequence data are available from the GENE EXPRESSION OMNIBUS (GEO) database, Genome Sequence Archive (GSA) database under the accession numbers GSE58915.
