# Supplementary material for: The mechanism of Vicenin-2 in ameliorating skin photoaging: involvement of m6A-modified macrophage polarization
Source: Front Pharmacol. 2026 Feb 19;17:1778993. doi: 10.3389/fphar.2026.1778993 (PMC12960571; doi:10.3389/fphar.2026.1778993)
Supplement: Supplementary file 1 [file Table1.docx]

Table S1. Data of relevant indicators for each group

| Group | Epidermal thickness (μm) | Skin moisture content (%) | Spleen index (%) | Collagen fiber (%) | Collagen I | Collagen III | Collagen I/III |
| --- | --- | --- | --- | --- | --- | --- | --- |
| Control | 21.49 ± 2.37 | 71.29 ± 1.31 | 0.24 ± 0.05 | 31.75 ± 4.63 | 6.30 ± 0.71 | 0.41 ± 0.09 | 15.49 ± 1.81 |
| Model | 62.91 ± 5.89 | 44.95 ± 2.45 | 0.78 ± 0.02 | 12.05 ± 1.86 | 2.53 ± 0.59 | 0.37 ± 0.03 | 6.88 ± 2.09 |
| Blank cream | 18.67 ± 0.79 | 68.99 ± 2.04 | 0.30 ± 0.10 | 35.99 ± 1.29 | 5.80 ± 1.99 | 0.37 ± 0.15 | 16.24 ± 1.44 |
| Blank cream+UV | 51.99 ± 5.64 | 43.37 ± 4.17 | 0.66 ± 0.04 | 10.15 ± 2.95 | 2.55 ± 0.88 | 0.35 ± 0.06 | 7.67 ± 4.13 |
| 0.025% Vicenin-2 | 55.11 ± 4.89 | 42.89 ± 2.98 | 0.80 ± 0.03 | 16.88 ± 3.75 | 3.52 ± 0.35 | 0.41 ± 0.05 | 8.70 ± 0.96 |
| 0.05% Vicenin-2 | 29.67 ± 2.43 | 57.46 ± 1.57 | 0.41 ± 0.07 | 26.88 ± 5.01 | 5.84 ± 0.58 | 0.54 ± 0.19 | 11.65 ± 3.18 |
| 0.1% Vicenin-2 | 22.37 ± 2.54 | 70.77 ± 1.69 | 0.25 ± 0.13 | 36.16 ± 4.95 | 6.02 ± 1.03 | 0.41 ± 0.02 | 14.77 ± 2.27 |

*Footnotes:* Mean ± SD for continuous variables.

| Group | M1/M0 | M2/M0 | NF-κB p65/β-actin | Phospho-NF-κB p65/β-actin | KIAA1429/β-actin | m6A level |
| --- | --- | --- | --- | --- | --- | --- |
| Control | 0.14 ±0.06 | 0.33 ±0.06 | 0.72 ± 0.06 | 0.09 ± 0.01 | 0.17 ± 0.02 | 1.00 ± 0.00 |
| Model | 0.56 ±0.09 | 0.10 ±0.04 | 0.73 ± 0.04 | 0.44 ± 0.15 | 0.05 ± 0.01 | 0.39 ± 0.05 |
| 0.025% Vicenin-2 | 0.64 ±0.10 | 0.07 ±0.02 | 0.71 ± 0.01 | 0.44 ± 0.09 | 0.09 ± 0.03 | 0.52 ± 0.01 |
| 0.05% Vicenin-2 | 0.21 ±0.10 | 0.25 ±0.12 | 0.71 ± 0.03 | 0.28 ± 0.08 | 0.14 ± 0.03 | 0.79 ± 0.03 |
| 0.1% Vicenin-2 | 0.08 ±0.02 | 0.47 ±0.02 | 0.68 ± 0.04 | 0.10 ± 0.02 | 0.19 ± 0.04 | 1.02 ± 0.04 |

Table S2. Data of relevant indicators for each group

*Footnotes:* Mean ± SD for continuous variables.
